# A scheme to underpin key mediator(s) in Salinosporamide(s) against pan-tumor via systems biology concept

**DOI:** 10.1186/s12967-024-05299-0

**Published:** 2024-05-24

**Authors:** Ki-Kwang Oh, Sang-Jun Yoon, Seol Hee Song, Jeong Ha Park, Jeong Su Kim, Min Ju Kim, Dong Joon Kim, Ki-Tae Suk

**Affiliations:** Institute for Liver and Digestive Diseases, College of Medicine, Hallym University, Chuncheon, 24252 Korea

**Dear editor,**

During the last decade, Salinosporamide A (SSAA) isolated by *Salinispora tropica*, or *Salinispora arenicola* known as the p26 proteasome inhibitor (anticancer agent) led to a renaissance in marine natural products’ niche. Despite the highly value, the establishment of its therapeutic mechanism is still in a maze due to the lack of the supply from marine organisms. We postulated that the key SSA(s), target(s), and mechanism(s) can be confirmed with the help of database-driven analysis, including computer screening tools. Hence, the aim of this study was to underpin the key Salinosporamide (SSA) in 11 SSAs with systems biology concept. A report demonstrated that SSAs exert potential capability to dampen cancer cell in structure–activity relationship studies (SARS) [1]. It is intriguing structure to develop schemes that would make these molecules attainable in the laboratory via total synthesis, which consists of gamma-lactam-beta-lactone bicyclic scaffold (Fig. 1A). The intent of this study was to pioneer the uppermost SSA(s) in terms of conceptual cancer treatment with a devised platform (Fig. 1B).

**Fig. 1:**
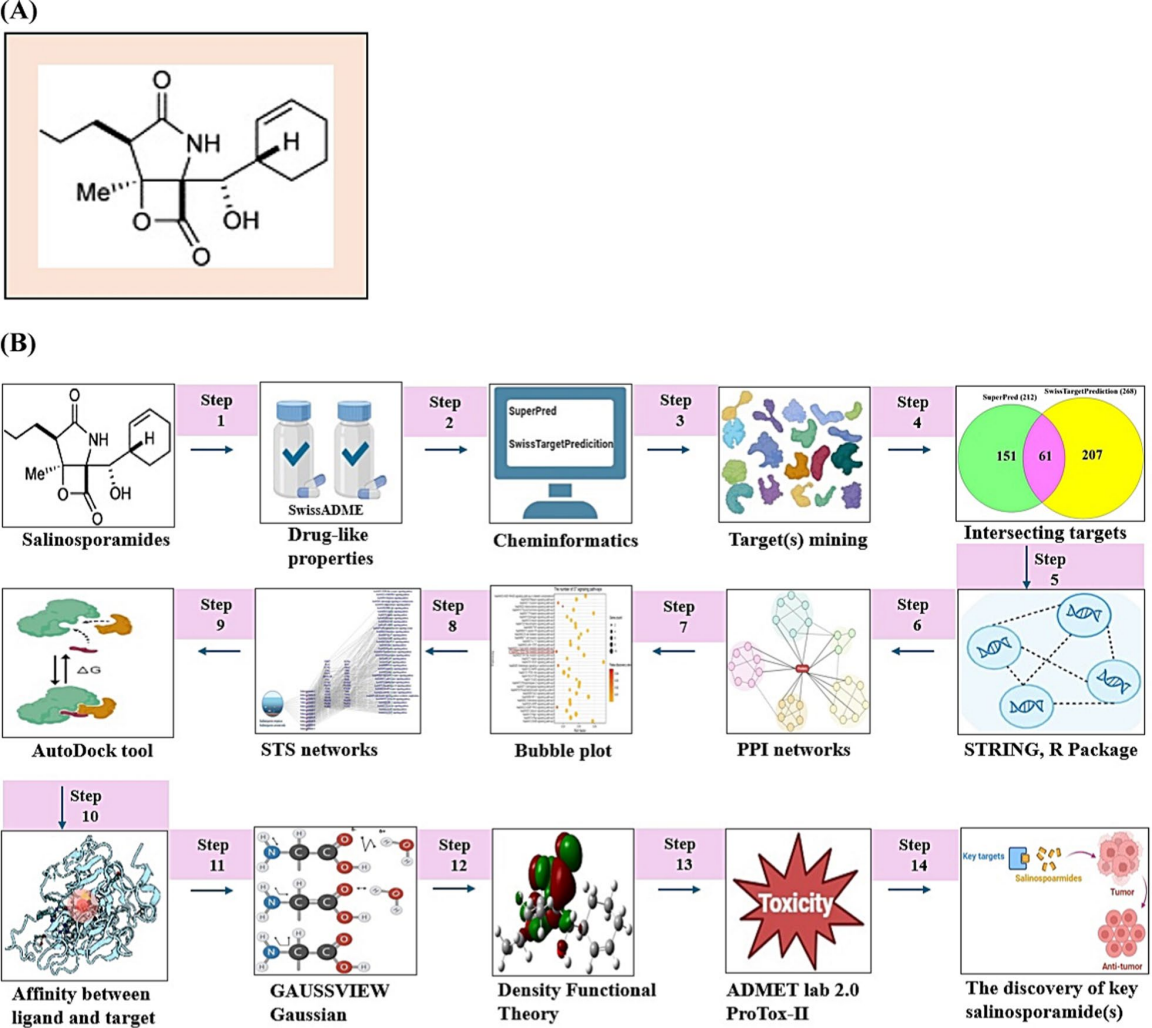

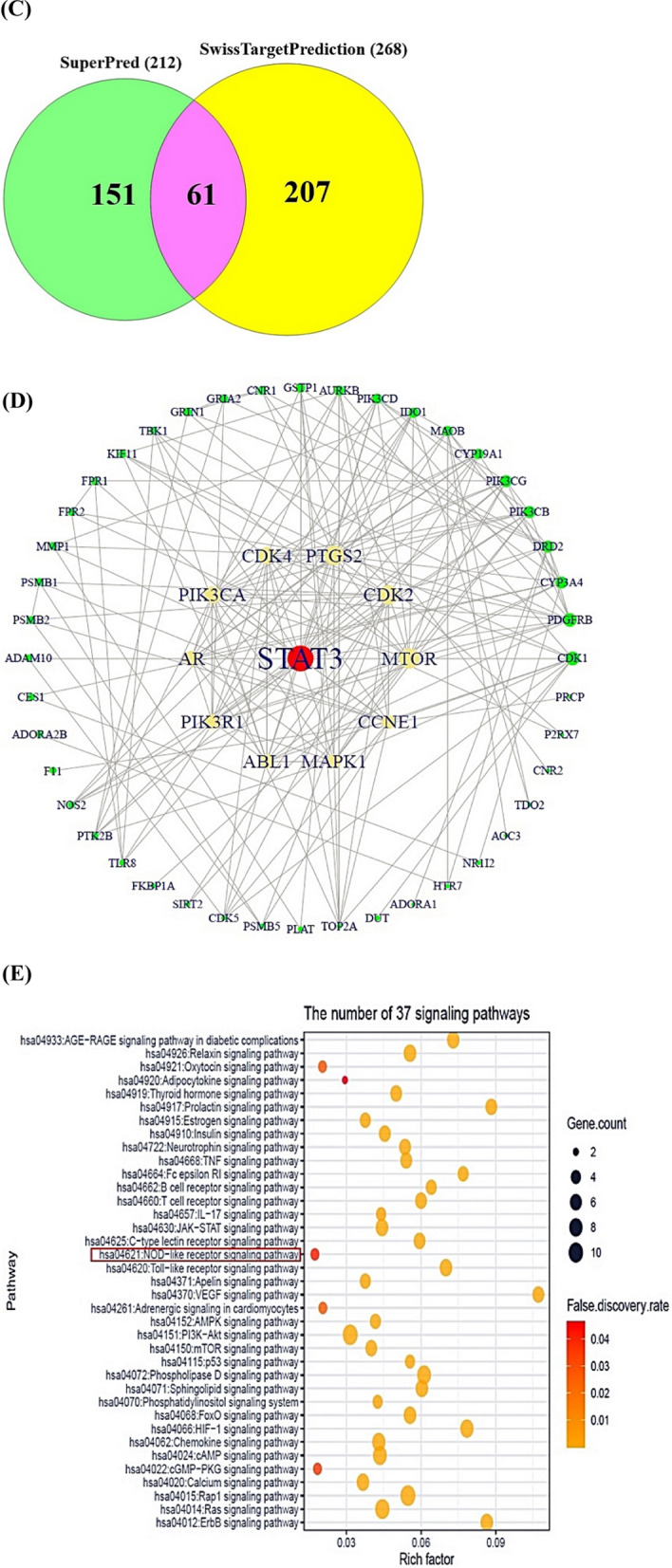

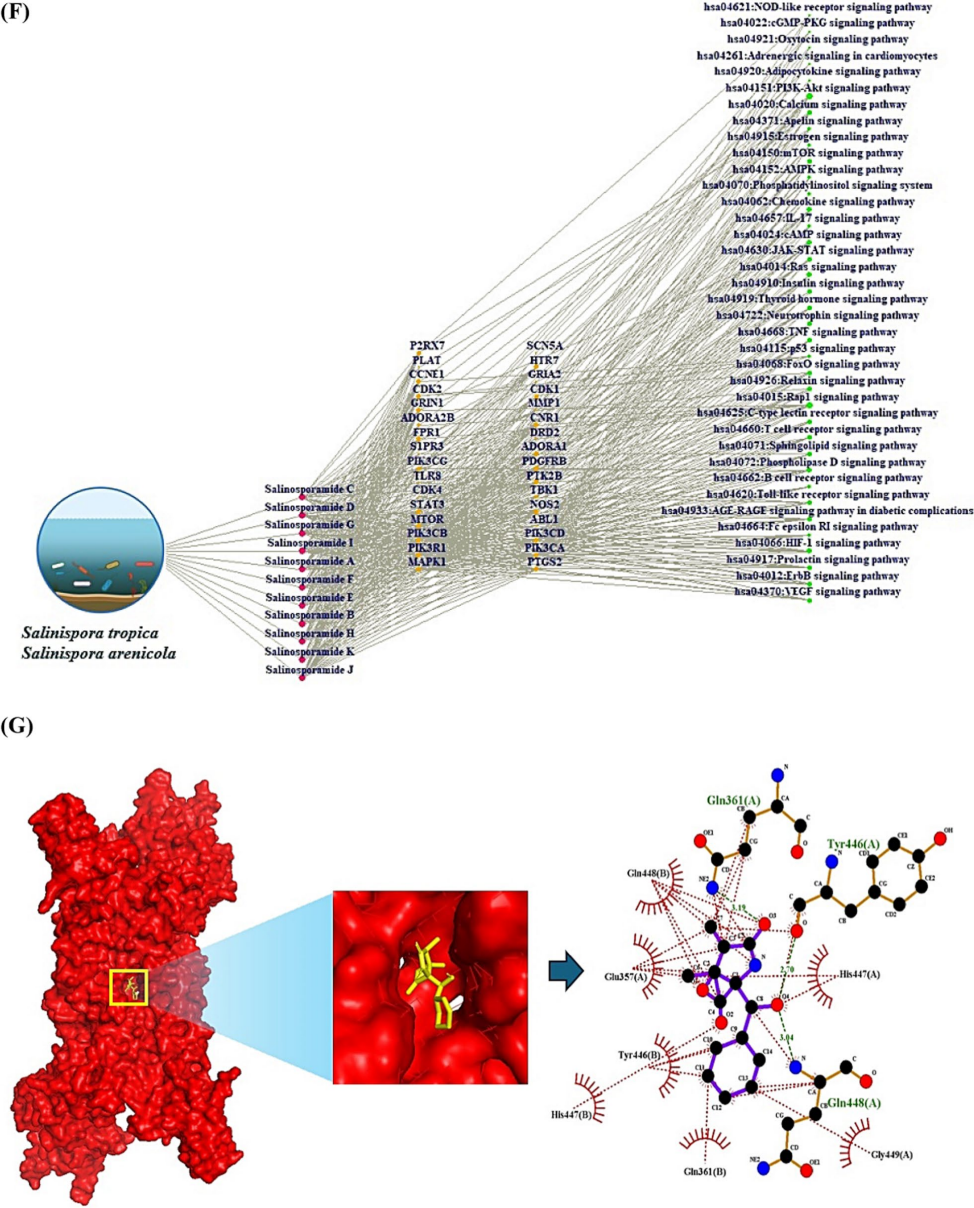

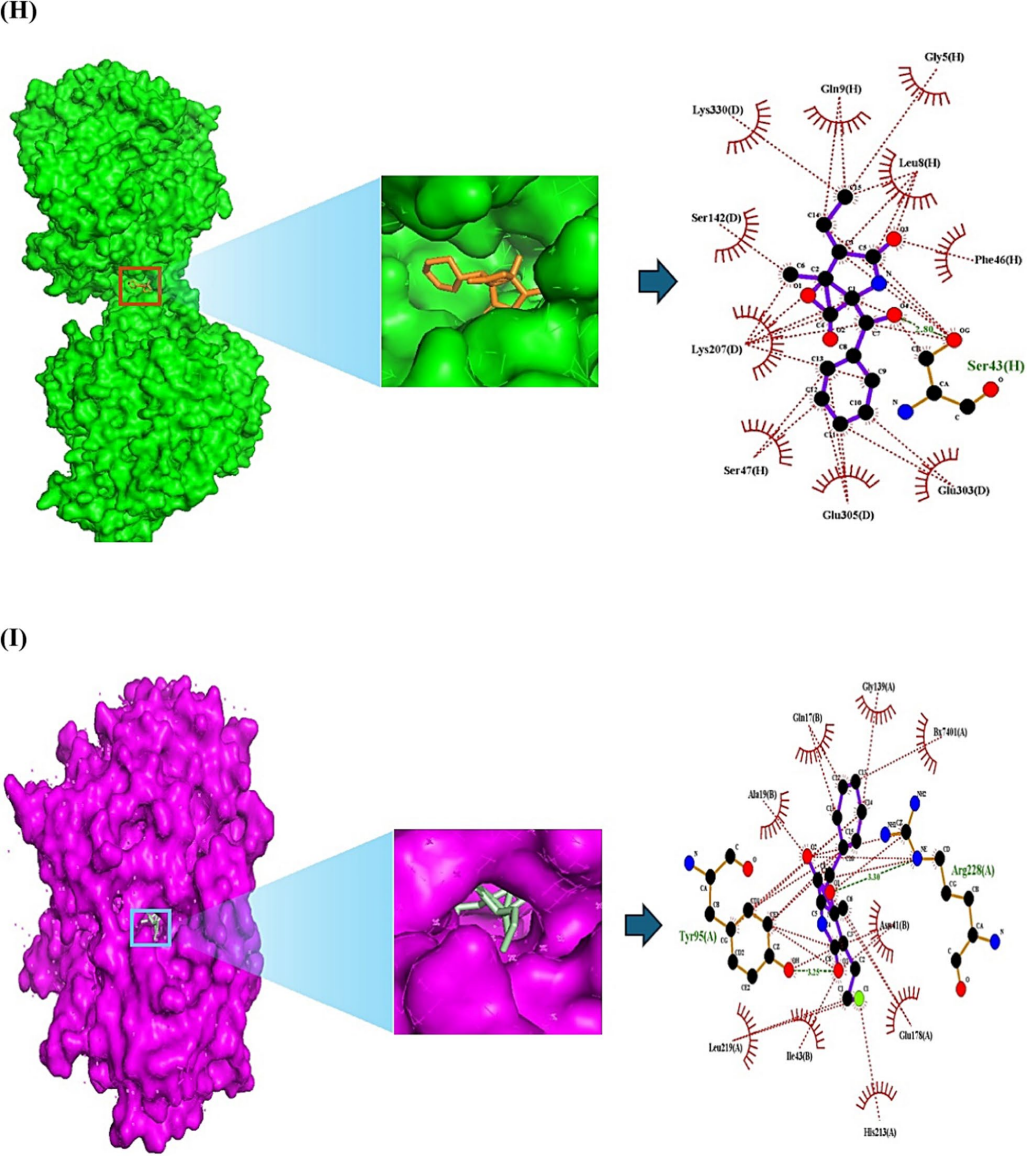

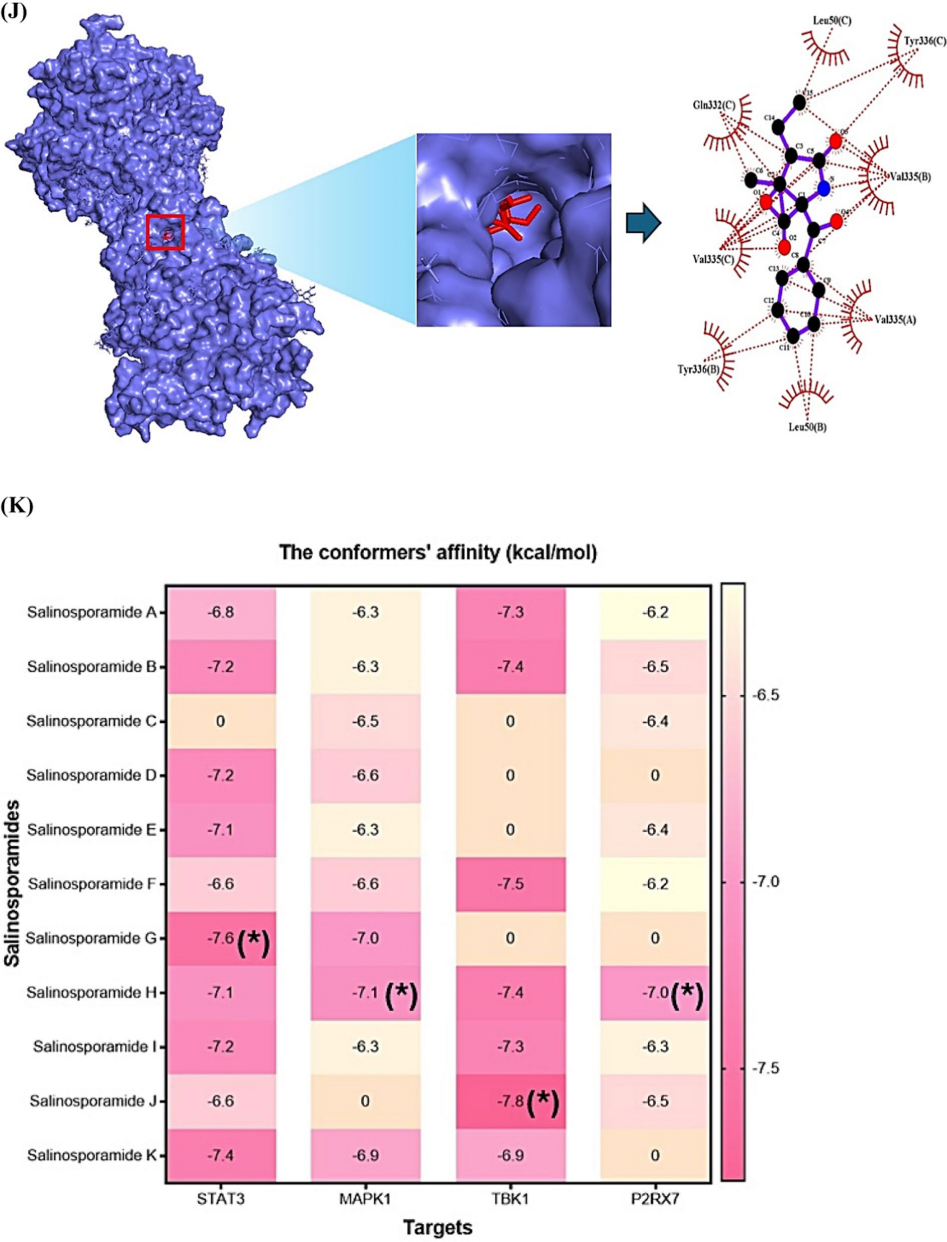

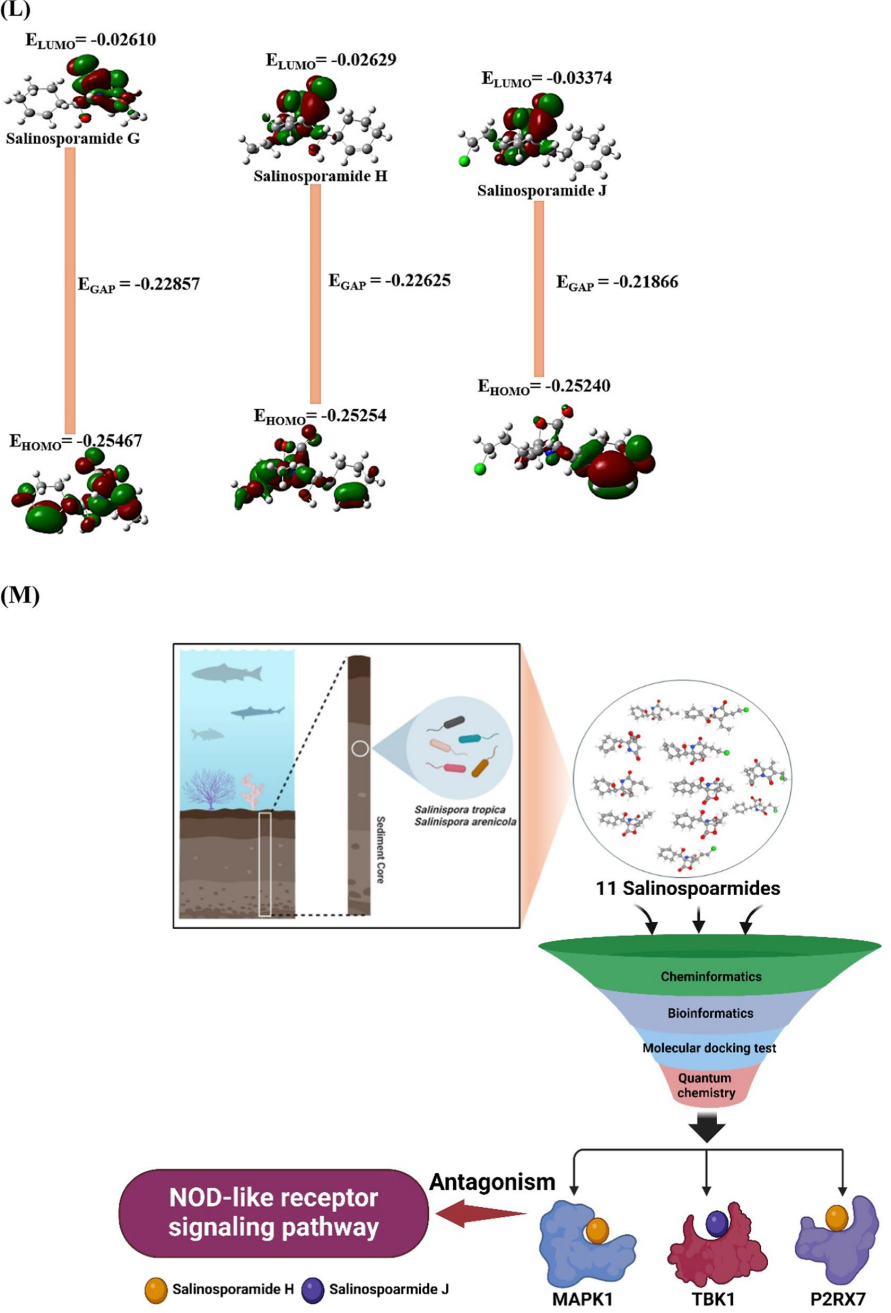
**A** The scaffold of Salinosporamides. **B** The stepwise of workflow for the study. **C** The overlapping targets (61) between SuperPred (212) and SwissTargetPrediction (268). **D** The protein–protein interaction (PPI) networks. **E** A bubble plot of 37 signaling pathways related to Salinosporamides against cancer. **F** The Salinosporamides-Targets-Signaling pathways (STS) networks. **G** The STAT3-Salinosporamide G conformer. **H** The MAPK1-Salinosporamide H conformer. **I** The TBK1-Salinosporamide J conformer. **J** The P2RX7-Salinosporamide H conformer. **K** The conformer’s affinity of four targets and three Salinosporamides. (*): The most stable conformer. **L** The energy gap (E_GAP_) of three key Salinosporamides via density functional theory (DFT). **M** The key summary of this study

First, the 11 SSAs were gathered through PubChem, and SwissADME platform was adopted to identify drug-like properties. All SSAs were accepted by the rigor criteria, suggesting that they can have potentiality to develop as therapeutic agent for human disease (Supplementary Table S1). Second, the common targets (61 targets) were selected between SuperPred (SP; 212 targets) and SwissTargetPrediction (STP; 268 targets), (Fig. 1C), (Supplementary Table S2). The number of 61 targets was considered as important protein-coding genes on SSAs against pan-tumor. Thereby, the protein–protein interaction (PPI) networks (55 nodes, and 198 edges) based on STRING database indicate that signal transducer and activator of transcription 3 (STAT3) is an uppermost target to modulate each target (Fig. 1D). Significantly, a previous study demonstrated that STAT3 inhibitors are key mediators on NOD-like receptor signaling pathway as antagonism [2]. A bubble plot to manifest the key signaling pathways was constructed by STRING, and R Package. Consequently, the 37 signaling pathways was linked to etiology of cancer (Supplementary Table S3). The NOD-like receptor signaling pathway with the lowest enrichment factor in them was identified an antagonistic mechanism on SSAs against cancer, indicating that the mechanism is a key mode in this study (Fig. 1E). In parallel, the corresponding targets on this mechanism were MAPK1, TBK1, and P2RX7. Holistically, 11 SSAs were associated directly with 32 targets, and 37 signaling pathways, which was constructed with Salinosporamides-Targets-Signaling pathways (STS) networks (81 nodes, and 482 edges) (Fig. 1F). Third, the identified targets with the 11 SSAs were docked to investigate the most stable conformers via Autodock tools. Above mentioned, STAT3-Salinosporamide G in PPI networks (Fig. 1G), MAPK1- Salinosporamide H (Fig. 1H), TBK1- Salinosporamide J (Fig. 1I), and P2RX7-Salinosporamide H (Fig. 1J) formed the most stable complexes in NOD-like receptor signaling pathway, Collectively, their affinity (threshold < − 6.0 kcal/mol) on each conformer was represented in Fig. 1K. Lastly, the density functional theory (DFT) was performed to validate the chemical reactivity of the SSAs (Fig. 1L), suggesting that E_GAP_ (Highest Occupied Molecular Orbital; HOMO—Lowest Unoccupied Molecular Orbital; LUMO), Hardness (ɳ; LUMO—HOMO/2), Softness (S; 1/ ɳ), and Electronegativity (χ;—(LUMO—HOMO)/2) of four SSAs are key parameters to confirm the chemical reactivity level. In the parameters, the range of S on anticancer agents was from 6.228 (eV) to 16.77785 (eV), including Vincristine, and Vinblastine [3]. The S value of key three SSAs was between 8.750 (eV) and 9.147 (eV), suggesting that they have valid chemical reactivity level to be anticancer drugs (Supplementary Table S4). Lastly, we confirmed the toxicity of the three key SSAs (Salinosporamide G, Salinosporamide H, and Salinosporamide J) via ADMET 2.0 and ProTox-II, thereby, they had no distinct hurdles to become new therapeutic mediator (Supplementary Table S5).

This study unlocks the therapeutic desirability of SSAs with underlying mechanism against cancer. The STAT3-Salinosporamide G in PPI network, the MAPK1-Salinosporamide H conformer, the TBK1-Salinosporamide J conformer, and the P2RX7-Salinosporamide H conformer were impactful epitome on NOD-like receptor signaling pathway in treating cancer. In conclusion, we propose that logical incorporation of the myriad biological data can contribute to decrypt the encoded anti-cancer agents, especially, illuminating marine organic molecules in limited amount is invaluable to cancer medicine. The key summary of this study was described in Fig. 1M.

### Supplementary Information

Supplementary Material 1.

Supplementary Material 2.

Supplementary Material 3.

Supplementary Material 4.

Supplementary Material 5.

## Data Availability

All data generated or analyzed during this study are included in this published article (and its Additional files).

## Abbreviations

DFT: Density functional theory
HOMO: Highest occupied molecular orbital
LUMO: Lowest unoccupied molecular orbital
PPI: Protein–protein interaction
S: Softness
STS: Salinosporamides-targets-signaling pathways
SARS: Structure–activity relationship studies
SP: SuperPred
SSA: Salinosporamide
SSAA: Salinosporamide A
SSAs: Salinosporamides
STAT3: Signal transducer and activator of transcription 3
STP: SwissTargetPrediction
χ: Electronegativity
